# Flow structure of okra mucilage in rotating wall vessel system

**DOI:** 10.1016/j.heliyon.2024.e36149

**Published:** 2024-08-21

**Authors:** Weijun Zhu, Hiromichi Obara

**Affiliations:** Tokyo Metropolitan University, Minamiosaw 1-1, Hachioji 192-0397, Tokyo, Japan

**Keywords:** Okra mucilage, Rotating wall vessel, Particle image velocimetry, Microparticle, Rheology, Spheroid incubation

## Abstract

The rotating-wall vessel (RWV) bioreactor, a 3D suspension culture system, faces challenges related to non-uniform tissue growth during the incubation of bone and heart tissues. Okra mucilage, an extract from okra pods with non-Newtonian rheological properties, has shown potential as a plasma replacement agent and has no induced cytotoxic effects. In this study, we investigated the flow structure of okra mucilage in rotating wall vessel system. By modifying the RWV and adding okra mucilage, we analyzed the flow structure using a high-speed camera and particle image velocimetry (PIV). Our results showed that okra mucilage creates a concentric circle-shaped rigid-like rotation at all rotation speeds (1-50 rpm). The high viscosity of okra mucilage resulted in a low terminal velocity for microparticles and quick response to rotational movements. These findings suggest that okra mucilage has the potential to enhance the uniformity of tissue growth in RWV systems by stabilizing the flow structure and reducing microparticle sedimentation.

## Highlights

• Okra mucilage in RWV displays the flow structure of rigid-like rotation (1–50 rpm).
• Microparticles will flow at low terminal velocity in the okra mucilage RWV system.
• In the initial stage, okra mucilage responds quickly to rotational movement.
• Weak elastic reversed flow is observed in okra mucilage when RWV suddenly stops.

## Graphical abstract

**Figure:**
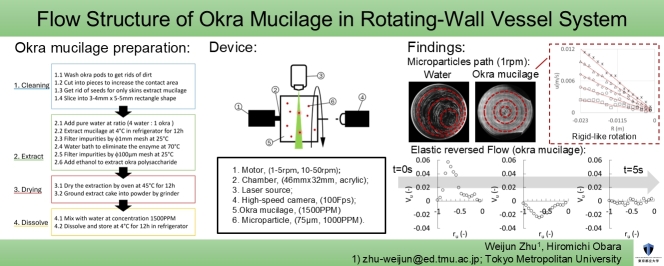

## 1 Background

Recently, the 3D cell culture method has been developed and is widely used. The cell incubation method in two-dimensional (2D), which involves growth on a flat substrate in a static environment, struggles to accurately replicate in vivo conditions, such as that in the human body. For example, in 2D, the growth of cells cannot be represented accurately when they are affected by disease and injury. Thus, limitations exist in the use of 2D cell culture for representing tissue cells in vitro [1]. However, cell growth in a 3D environment is a better method of representing human tissues outside the body. It allows cells to be more physiologically relevant and predictive during the incubation based on the in vivo morphology, cell connectivity, polarity, gene expression, and tissue structure [2]. Spheroid incubation, in which cells simply stick to each other and aggregate, is one of the most common methods used in the 3D cell culture. For stem cells, the spheroid incubation system can rebuild the biological signaling pathways of cell–cell, cell–ECM interactions that will promote cell proliferation and viability [3]. In addition to stem cells, embryoid bodies, neurospheres, hepatospheres, mammospheres, and multicellular tumor spheroids have also been reported successively under the spheroid incubation method [4], [5].

The RWV bioreactor was designed by the National Aeronautics and Space Administration (NASA) to model microgravity. It can be described as a slow-turning lateral vessel that rotates on a platform. The RWV bioreactor provides a simulated microgravity environment, leading to a low fluid shear environment for cells. A constant free fall environment under low fluid shear, wherein the cells can attach to and grow on beads, helps to form visible cellular aggregates [6]. It has been found that the RWV system can more precisely present the features of human tissues in vivo [7]. Therefore, the RWV bioreactor will be a valuable device. It holds promise for testing the efficacy, toxicity, and pharmacokinetics of vaccines, microbicides, biologics, and drugs [6], [8], [9]. In addition, the RWV system has recently been extended to tissue engineering, regenerative medicine, and tumor modeling [6], [10].

Okra is a seedpod that comes from the *Abelmoschus esculentus* plant, which belongs to the mallow family. It can be eaten as a vegetable and purchased from the market in daily life. Okra pods are rich in proteins, fats, carbohydrates, calcium, phosphorus, iron, and many other vitamins. It makes okra pods a beneficial dietary source for the human body. When okra pods are sliced and cooked, they secrete a thick and viscous liquid known as okra mucilage. Okra mucilage has been widely used and researched in rheology, the food industry, pharmacology, and biomedical applications. The main compound of okra mucilage is okra mucilage polysaccharides. The okra mucilage polysaccharides are found to have a random coil structure consisting of galactose, rhamnose, and galacturonic acid [11]. The characteristics of good biocompatibility and high availability in nature have been demonstrated in a study on okra mucilage [12]. None of the induced cytotoxic effects is found in vitro and in vivo toxicity studies on the use of okra mucilage in mice [13]. Okra mucilage is a safe pharmaceutical excipient. It can be successfully used to create modified-release formulations [14], [15], [16]. In addition, okra mucilage reduces gastric injuries, lipid peroxidation, ulcerated areas, edema, hemorrhage, cell infiltration, and epithelial cell loss and improves antioxidant defense systems [17]. In drug delivery systems, okra mucilage can act as a binder, suspending agent, floating agent, film coating agent, plasma expander, an anti-adhesive, emulsifying agent, a stabilizer, thickener, or an antioxidant [18]. Okra mucilage extracts are strong candidates for emulsification in acidic environments [19]. This property can be exploited to deliver emulsified hydrophobic drugs or nutrients through the acidic environment of the stomach [20]. In terms of rheological properties, okra mucilage has been proven to be a structured fluid having viscoelastic, shear-thinning, adhesive, stringy, ductile, and cohesive properties [21]. The weak gel behavior of okra mucilage is also observed [22]. The extensional viscosity of okra mucilage significantly exceeds its shear viscosity (by two or three orders of magnitude) [23]. Its viscous and elastic modulus decrease after the shear history effect [24].

The shear thinning properties of culture mediums can mitigate shear force damage to cells within a specific range, enhancing cell viability and proliferation rates [25]. The viscoelastic characteristics of the culture medium influence the mechanical stimulation environment of cells by altering its viscosity and elasticity, thereby impacting cell differentiation and functional expression [26]. Additionally, shear history effects and extensional viscosity contribute to the complexity of fluid dynamics, significantly affecting the cell growth environment. In the context of rotating wall vessel (RWV) systems, previous research has shown that medium viscosity is crucial. Higher viscosity can prevent denser microcarriers from reaching the outer wall of the vessel, where cell damage can occur [27], [28], [29]. The viscosity of the culture medium is a critical parameter in RWV bioreactors, affecting cell growth and metabolic efficiency. Studies have demonstrated that higher viscosity can provide a more stable suspension state, promoting better cell growth and functional performance in bone marrow cell cultures [30]. Regarding fluid dynamics behavior, the viscosity and density of the medium determine the movement and suspension state of particles in the RWV bioreactor. Proper adjustment of these properties can optimize the suspension and distribution of cells, enhancing culture efficiency [31].

Currently, the RWV is encountering challenges related to non-uniform tissue growth during the incubation of bone and heart tissues. The RWV needs control systems to vary the rotation speed of the vessel in function of the tissue size [32]. In our study, we aim to explore how the rheological properties of okra mucilage might contribute to improving tissue growth uniformity in the RWV system. The first step of our study is to explore the flow structure of okra mucilage in the RWV system. The flow structure information was obtained using a high-speed camera and analyzed with particle image velocimetry (PIV). Furthermore, the sediment velocity of microparticles in okra mucilage was tested, as well as the initial and final stages of microparticles flowing in the RWV. Our research shows that okra mucilage creates a stable flow structure with rigid-like rotation for microparticles in the RWV system at speeds ranging from 1 to 50 rpm. And the addition of okra mucilage will decrease the terminal velocity of the microparticles. In the initial stage, okra mucilage responds quickly to rotational movement. On the other hand, weak elastic reversed flow happens in okra mucilage when the RWV system suddenly stops.

## 2 Method and materials

### 2.1 Extraction of okra mucilage

**Figure 1:**
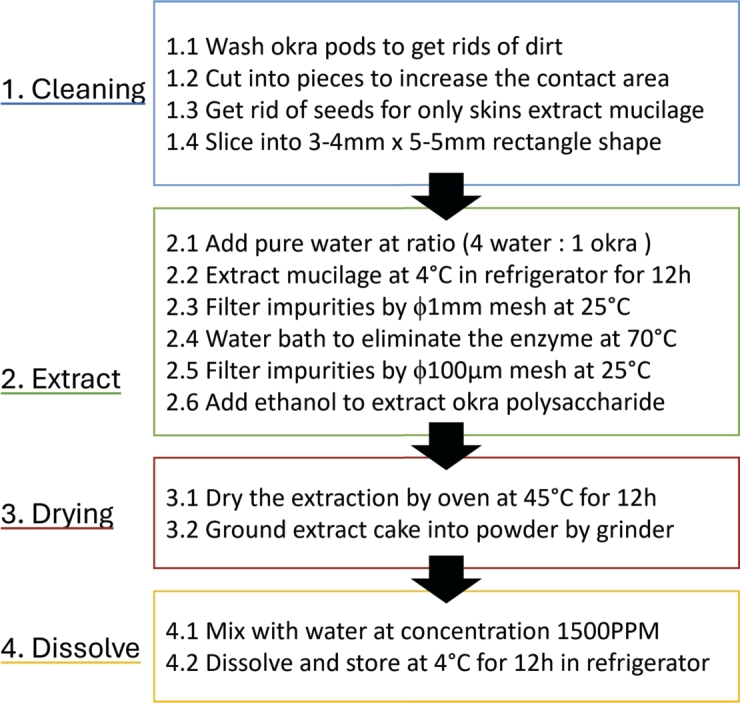
Protocol of okra mucilage preparation.

Fresh okra pods were obtained from a local market and used to extract the okra mucilage. The method used to secrete okra mucilage was described in a previous study [24]. After the secrete process, the slightly yellow and thick okra mucilage was collected in a beaker for a water bath at 70 ^∘^C. This step is performed to let okra endogenous enzymes inactivate and contribute to the stability of the okra mucilage powder during storage. Then the okra mucilage was cooled to room temperature. A smaller mesh (100 μm) was used to remove insoluble materials from the okra mucilage. Four times the volume of ethanol was mixed with okra mucilage to create a flocculent extraction. The flocculent was filtered and dried in an oven at 45 ^∘^C for 12 h. The dried okra mucilage extraction cake was placed in a grinder and manually ground into powder for stable and easy storage. To prepare the okra mucilage before the experiment, the okra mucilage extraction powder was dissolved in pure water. The okra mucilage was stored in the refrigerator at 4 ^∘^C for 12 hours until the powder dissolved completely in pure water. After recovering the okra mucilage to room temperature, the experiments were initiated. The concentration of okra mucilage was set at 1500 ppm. The density of the okra mucilage was tested and found to be 1.01 g/cm^3^. The simplified protocol of okra mucilage preparation is shown in Fig. 1.

### 2.2 Control group and microparticle

We chose water as a Newtonian control group because cell culture mediums are often aqueous solutions. An artificial, chemical substance, polyethylene oxide 15 (PEO15, MW: 3.3 - 3.8×10^6^ g/mol) powder, was selected as the control group, too. In experiments, the concentration was also set as 1500 ppm. The solution PEO15 (1500 ppm) exhibits a viscosity similar to that of okra mucilage (1500 ppm) at high shear rates. The density of water and the PEO15 solution are 0.99 g/cm^3^ and 1.05 g/cm^3^, respectively.

The reddish-pink, fluorescent microspheres from Dan Tec Co., Ltd. were used in a series of experiments. Given that hepatocytes (20 - 30 μm) aggregate to form a diameter of 70 μm, we chose the microparticle diameter to be 70 μm. The microparticles in all the samples were set to a concentration of 1000 ppm, at which, the microparticles were easy to observe. The density of the microparticles is 1.10 g/cm^3^.

### 2.3 Rotation experiments

**Figure 2:**
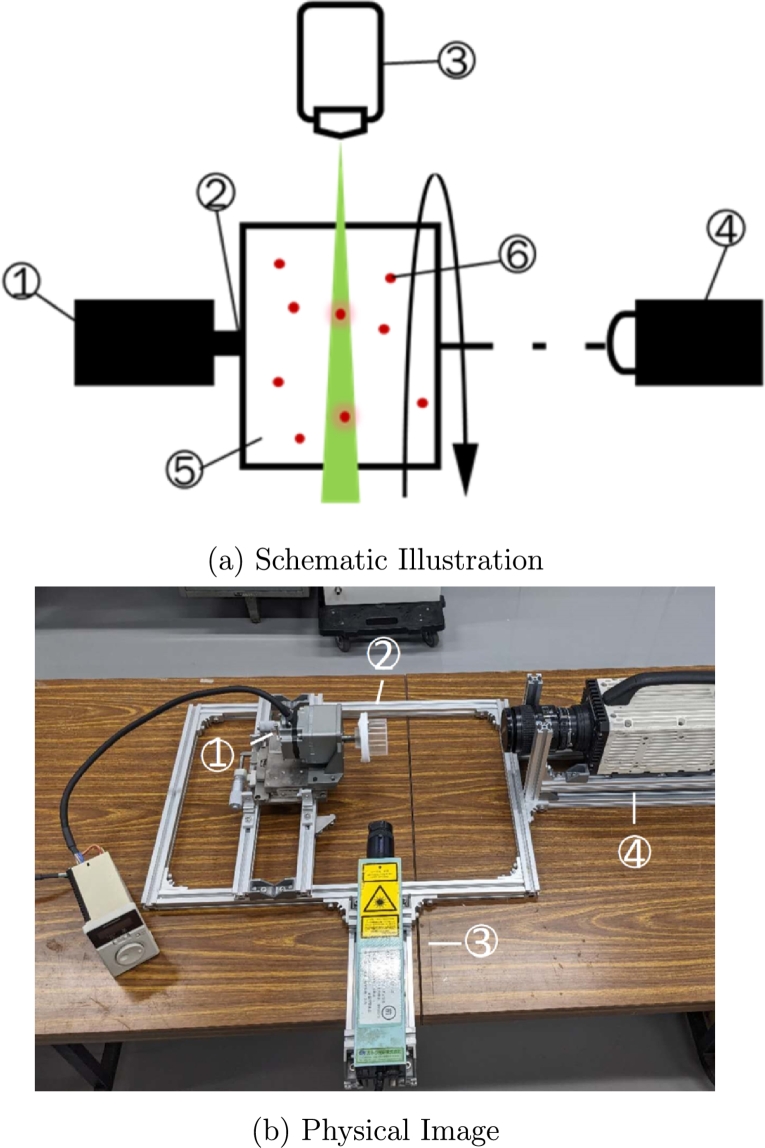
(a): Schematic illustration of experiment device; (b): Physical image of experiment device. (1. Rotation motor (Oriental motor, PKP523N12A), 2. Cylinder chamber (46x32 mm), 3. Laser source (Kadou Hikari), 4. High speed camera (NAC, Memrecam GX1-F), 5. Water/PEO15 solution/Okra mucilage, Microparticle (75 μm)).

The RWV chamber for experiments comprised a cap, base cylinder roller, sealing ring, and some screws. It was designed and assembled by us. The cap and base cylinder roller were made of transparent acrylic board (thickness: 1 mm) so that the laser light could pass through. The inner diameter of the chamber was 46 mm, and its height was 32 mm. The total capacity volume of the chamber was about 53 ml. The experiment solution was added to the chamber base, and the cap was closed, ensuring there were no air bubbles in the chamber. Then, the chamber would be installed on a rotation motor (Oriental Motor, PKP523N12A). In cell incubation, rotation speed is adjusted for different cell types, but is typically lower than 50 rpm. Thus, the motor was rotated at low speeds of 1-5 rpm and high speeds of 10-50 rpm. The laser source from Katou Co. Ltd. was positioned to scan the middle cross-section of the cylindrical chamber. After running for 5 minutes, the flow structure remained stable. Results were recorded for 10 s using a high-speed camera (NAC, Memrecam, GX-1F), positioned at the centerline opposite the cylinder chamber. The recording speed was set at 100 fps. The schematic image of entire system is illustrated in Fig. 2-a and physical image of experiment device is showed in Fig. 2-b.

### 2.4 Initial and final movements of microparticle

In the initial movement experiment, the chamber was placed on the motor and kept still for 5 minutes. Next, the motor was abruptly turned on to 10 rpm. The high-speed camera recorded the initial movement of the microparticles for the first 10 s after the motor was turned on.

During the final movement experiment, the motor was set to run at 50 rpm. Once the flow structure had become stable, the motor was turned off. At the same time, the high-speed camera began recording the movements of the microparticles in the solution. The recording lasted from 0 s to 10 s.

### 2.5 Sedimentation experiments

The sedimentation experiment was conducted after the final stage of the experiment. The RWV system was held steady for 60 s to eliminate the influence of radial tangential velocity due to inertia. The movements of the microparticles under gravity were then recorded using a high-speed camera for 10 s. The microparticles at the center of the chamber, which fell straight, were selected, and calculated for the sediment velocity.

### 2.6 Rheological property [24]

To conduct tests on the rheological properties of the solution, a HAAKE Rheostress 600 from Thermo Electron Corp was used. According to the HAAKE Rheostress 600 handbook tables, the cone C60/1 Ti was selected. The diameter, geometry angle, and gap were 60 mm, 1^∘^, and 0.052 mm, respectively. Wet tissues were placed around the plate to maintain the humidity and prevent rapid moisture loss from the okra mucilage and PEO15 solution, while all the rheological tests were performed. All the measurements were repeated at least three times.

### 2.7 Particle image velocimetry (PIV)

The iterative PIV program implements two image-correlation methods. The conventional cross-correlation method was used in this study. This is performed using the PIV macro program of the Fiji ImageJ application. Each PIV result serves as a window preshift for the next PIV iteration such that a large displacement can still be captured when a small interrogation window is used [33]. The data were collected and analyzed from multiple pictures captured using a high-speed camera. In these experiments, the velocities were converted to SI units using the ratio of pixels in the picture to the experiment chamber radius.

### 2.8 Terminal velocity

The microparticles in a RWV system accelerate until reaching terminal velocity, balancing the pull of gravity with hydrodynamic forces such as shear, centrifugal, and Coriolis. The shear force acting on the particles is proportional to the terminal velocity of the microparticles, which is in turn determined using Eq. (1) [34].

(1) $$V_{s}=\frac{2gr^{2}(\rho_{p}-\rho_{f})}{9\mu_{0}}$$

where $V_{s}$ is the terminal velocity, g is the gravity, r is the radius of the microparticles, $\rho_{p}$ is the density of the microparticles, $\rho_{f}$ is the density of the sample solution, and $\mu_{0}$ is the zero-shear viscosity of the sample solution.

### 2.9 Maximum shear stress

The maximum shear stress acting on the microparticles in the RWV is a function of the terminal velocity, viscosity of the solution, and diameter of the microparticles. It is defined by Eq. (2) [34].

(2) $$\tau_{max}=\frac{3\;{\mu \text{V}}_{s}}{2r}$$

where $\tau_{max}$ is the maximum shear stress, $V_{s}$ is the terminal velocity, g is gravity, and r is the particle radius.

### 2.10 Basic data of each sample

In rigid-body motion, the bulk flow moves without relative motion. Thus, the zero-shear viscosity of the PEO15 solution and okra mucilage were selected for calculating terminal velocity, maximum shear stress, and Reynolds number in this study.

The density (*ρ*), concentration (c), and zero-shear viscosity ($\mu_{0}$) of samples are listed in Table 1. For creeping flow around a solid sphere condition, the Reynolds number of microparticles in the RWV system is a dimensionless quantity obtained by using Eq. (3). It is equal to the ratio of inertial and viscous forces [35]. The Reynolds number of microparticles near the wall at 50 rpm is displayed in Table 1.

(3) $$Re=\frac{2\rho_{f}(V+V_{p})r}{\mu_{0}}$$

where Re is the Reynolds number, $\rho_{f}$ is the density of the sample solution, V is the velocity of the solution, $V_{p}$ is the velocity of the microparticles, r is the particle radius and $\mu_{0}$ is the zero-shear viscosity of the sample solution.

**Table 1:** Basic data of each material.

Material/Parameter	*ρ* (g/cm^3^)	c (ppm)	*μ*_0_ (Pas)	Re (50rpm)
Water	0.99	/	0.001	1.69E+01
PEO15 solution	1.05	1500	0.030	5.90E-01
Okra mucilage	1.01	1500	0.225	8.51E-02
Microparticle	1.10	1000	/	/

## 3 Results and discussions

### 3.1 Viscosity

**Figure 3:**
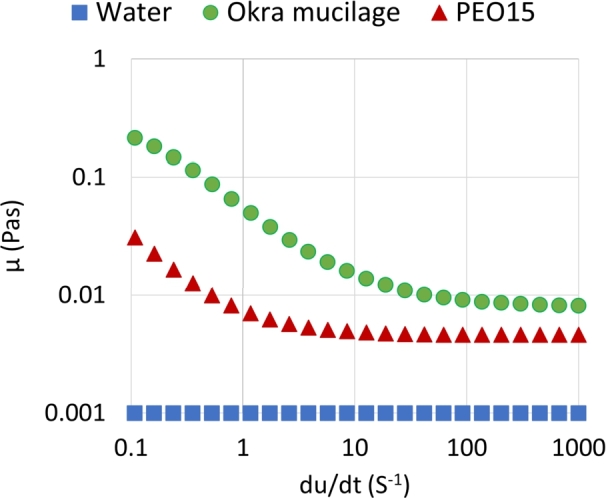
Viscosity as a function of shear rate of each sample.

Fig. 3 displays the viscosity of each sample as a function of the shear rate. Water is a Newtonian fluid, and its viscosity is constant at all shear rates. However, at the same concentration, the okra mucilage and PEO15 solutions exhibit a continuous decrease as the shear rate increases. They are shear-thinning liquids. At shear rates ranging from 0.1 ${\text{s}}^{-1}$ to 1000 ${\text{s}}^{-1}$, the viscosity of okra mucilage is highest, followed by PEO15, with water having the lowest value. At a shear rate of 1 ${\text{s}}^{-1}$, the viscosity of okra mucilage is approximately 0.057 Pas. As the shear rate increases to 1000 ${\text{s}}^{-1}$, the viscosity decreases to around 0.008 Pas due to shear thinning. The viscosity-shear rate profile of okra mucilage observed in this experiment closely matches the findings reported by Junfang Zhu [23]. It is also worth noticing that the previous research on the rheological properties of okra mucilage shows that the first normal stress difference is around 0.02 Pa, and the relaxation time is approximately 4.5 s [24].

### 3.2 Sediment velocity

**Figure 4:**
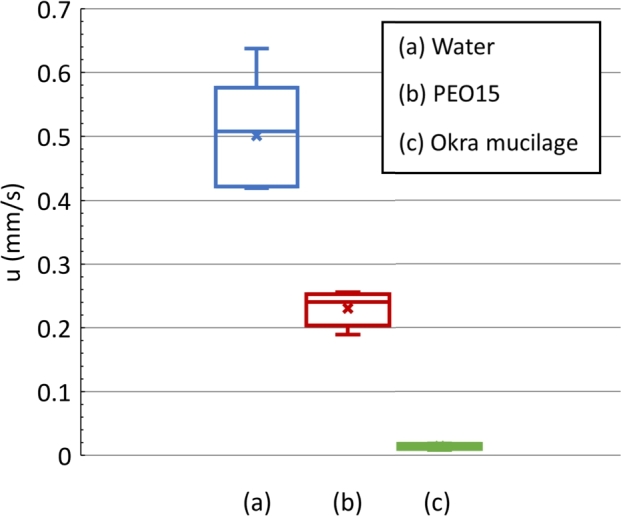
Sediment velocity of microparticles in each sample; (a): Water, (b): PEO solution, (c): Okra mucilage.

Fig. 4 presents the sediment velocity of the microparticles in each sample. The microparticles in water have the highest sediment velocity (Fig. 4-a). In contrast, the sediment velocity of the microparticles in the okra mucilage is the lowest (Fig. 4-c). The sediment velocity of the microparticles in the PEO solution falls in between these two samples (Fig. 4-b). The settling velocity of microparticles is affected by the viscosity and rheology of the solution due to the controlled radius of the particles. Viscosity is the resistance of a fluid to deformation forces caused by either shear or tensile stress. A higher viscosity results in a lower sedimentation efficiency [36]. The viscosity of okra mucilage is the highest, resulting in the lowest sediment velocity of microparticles.

### 3.3 Micropartilce path

**Figure 5:**
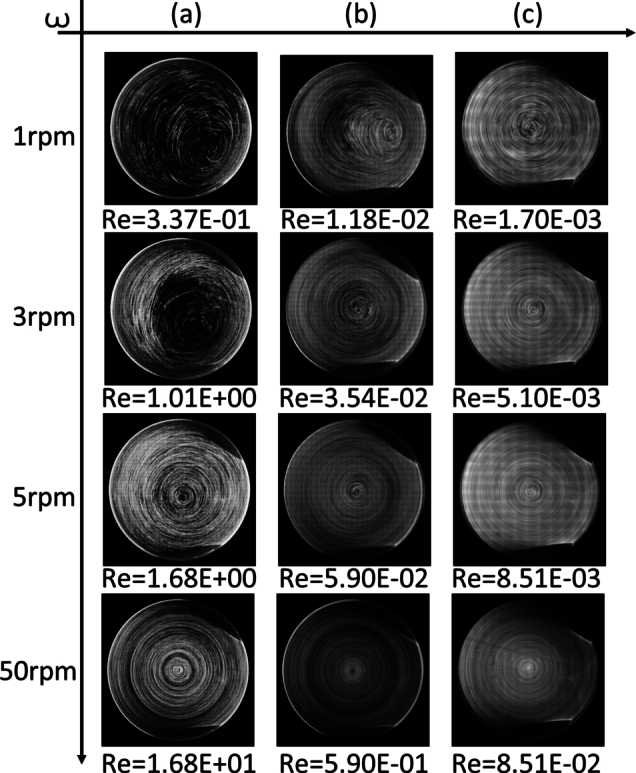
Particle path in each sample for various rotation speeds; (a): Water, (b): PEO solution, (c): Okra mucilage.

Fig. 5 shows the microparticle paths at rotating chamber speeds of 1, 3, 5, and 50 rpm for water (Fig. 5-a), PEO15 solution (Fig. 5-b), and okra mucilage (Fig. 5-c). When rotating at speeds below 3 rpm in water and 1 rpm in PEO solution, microparticles form an eccentric circular path. Afterwards, all microparticle paths become concentric circles. In contrast, the microparticle path in the okra mucilage remains stable with concentric circles from 1 to 50 rpm.

In the experiment, the rotation speed of the chamber, the density difference between the microparticles and liquid, and the microparticle size are controlled. The rotation of the RWV induces both Coriolis and centrifugal forces. When the rotational speed is not sufficiently high, the gravitational and Coriolis forces are dominant. The microparticles fall from the upper right area of the wall due to gravity and are affected by Coriolis forces, causing them to change direction. The path of the microparticle appears to follow an eccentric circular shape. As the rotation speed increases, the centrifugal effect becomes dominant. The combination of centrifugal force and confinement from the wall leads to the formation of concentric circles as the fluid moves away from the axis.

According to the Reynolds number, it can be concluded that the flow in all samples (1-50rpm) is laminar. In the steady flow, the shape of the flow structure is the same as the microparticle path. Thus, at rotation speeds of 1 to 50 rpm, the flow structure of okra mucilage is a concentric circle shape.

The low sediment velocity of microparticles in the okra mucilage represents that okra mucilage has a stable suspension property. This is because okra mucilage provides a low qualitative flocculation and high re-dispersible environment [37]. The combination of low flocculation and high re-dispersibility implies that the suspension is stable. The particles have a tendency to remain well-distributed in the liquid, and if settling occurs, it's reversible. In addition, okra mucilage is a hydrophilic polymer matrix suspension [20]. The polymer chains may form a protective layer around microparticles, preventing or reducing sedimentation. The high viscosity of okra mucilage also improves the fluid-carrying capacity [38]. This ensures the uniformity of the microparticles. At a low rotation speed regime, the paths of microparticles are not substantially influenced by gravity. Thus, the flow structure in the okra mucilage remains ordered and stable with concentric circles from 1 to 50 rpm.

However, to okra mucilage, if the rotation speed of RWV is high enough, the centrifugal effect will be large. This can result in more pronounced outward migration of microparticles towards the wall. Furthermore, if the rotation speed of RWV is pushed to limit, the fluid will be complex. Because it is not only dependent on viscous forces varied from shear-thinning, but also the influence of transition from laminar to turbulent flow and rheological properties such as shear history effect, shear-induced microstructures.

### 3.4 Particle image velocimetry (PIV)

**Figure 6:**
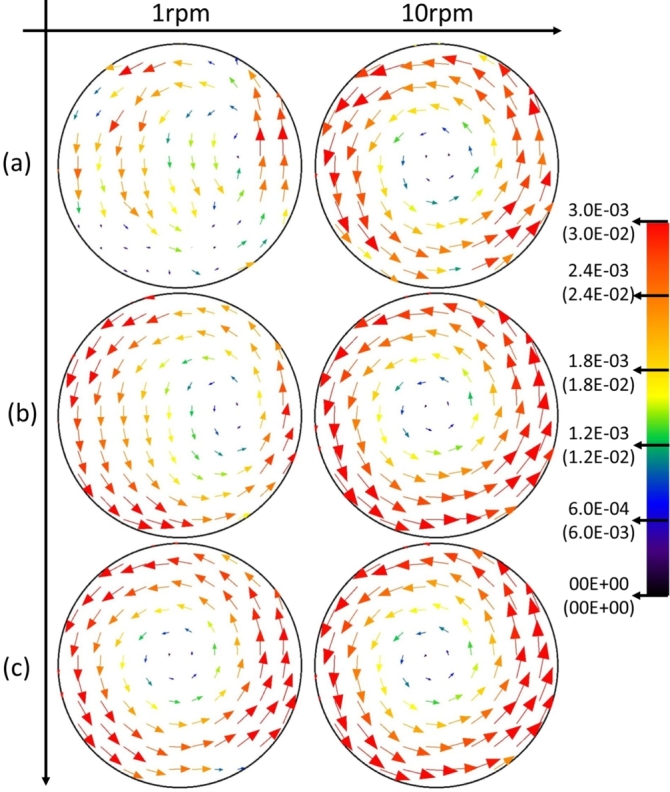
Velocity vector view of each sample in PIV for various rotation speeds; (a): Water, (b): PEO solution, (c): Okra mucilage.

The velocity vector view of the microparticles in the water (Fig. 6-a), PEO15 (Fig. 6-b), and okra mucilage (Fig. 6-c) is presented in Fig. 6. It shows that the tangential velocity of microparticles can vary depending on their radial distance from the axis of rotation. The microparticles closer to the center experience a lower tangential velocity, while the ones near the wall move with the same tangential velocity as the wall.

**Figure 7:**
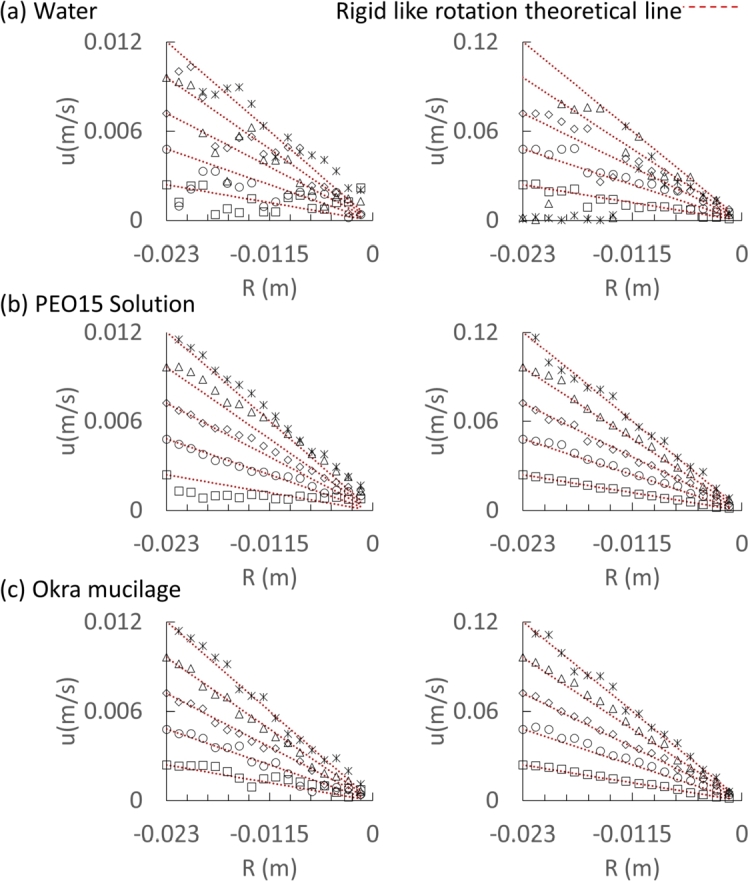
Velocity value as a function of the radius of each sample; (a): Water, (b): PEO solution, (c): Okra mucilage. (Left: □: 1rpm, ◯: 2rpm, ⋄: 3rpm, △: 4rpm, ×: 5rpm.; Right: □: 10rpm, ◯: 20rpm, ⋄: 30rpm, △: 40rpm, ×: 50rpm.)

To investigate the movements of the microparticles, the velocity as a function of the radius of each sample is presented in Fig. 7. In a rotation system, the rigid-like rotation shows that the tangential velocity varies linearly with the radial distance. In Fig. 7, the red dashed line is drawn and represents the rigid-like rotation. As shown in Fig. 7-a, in water, the microparticle velocity starts to close the proportional trend line from 4 rpm. When the rotation speed exceeds 30 rpm, the velocity of the microparticles near the wall loses speed. This is because, for water RWV systems, rotation speed over 30 rpm is sufficiently high. Moreover, microparticles near the wall experience stronger forces, while those closer to the center experience weaker centrifugal forces. Only microparticles near the wall migrate and attach towards the wall of the rotating cylinder first. It reduces the number of detectable microparticles, making PIV analysis difficult. In Fig. 7-b, when the rotation speed is higher than 1 rpm in the PEO15 solution, the rotation system turns into a rigid-like rotation. In Fig. 7-c, it is worth noticing that the velocity curves of the microparticles in okra mucilage at all experiment rotation speeds (1-50rpm) exhibited rigid-like rotation.

When the cylinder is rotating, the fluid experiences shear forces due to the velocity gradient across its layers. However, the high zero-shear viscosity of okra mucilage provides stability against shear. It prevents excessive deformation and maintains the integrity of the flow. Moreover, the nice suspensibility of okra mucilage contributes to the even distribution of forces within it. This uniform distribution is essential for maintaining a consistent flow structure, especially when the rotational speed is not high enough to induce unstable flow (gravity induced the eccentric circular shape flow). Thus, the microparticles in okra mucilage flow under a rigid-like rotation observed in Fig. 7 at rotation speeds ranging from 1 to 50 rpm

In RWV, the cells under gravity fall towards the vessel bottom and are brought upward owing to the rigid-like rotation of the liquid, which makes them suspend in an orbital path [39], [40]. The rigid-like rotation facilitates the simulation of a microgravitational environment, establishing conditions characterized by low shear and minimal turbulence for optimal cell culture [34], [41], [42]. In addition, the rigid-like rotation has the features of the colocalization of cells and aggregates with different sedimentation rates and reduced 3D spatial freedom [34]. This environment, created by rigid-like rotation, is suitable for organoid and spheroid generation.

### 3.5 Terminal velocity and Maximum shear stress

In the chamber, the cells flow in a rigid-like rotation without turbulence. The tangential relative displacement of different layers is low. Thus, the zero-shear viscosity of PEO15 solution and okra mucilage are selected for calculating terminal velocity and maximum shear stress. The velocity of microparticles is accelerated through the fluid until it reaches the terminal velocity, where the accelerated motion induced by gravity and the viscous drag of the liquid are balanced. In Table 2, the microparticles in water exhibit the highest terminal velocity among all the samples, while okra mucilage provides the lowest velocity for the microparticles. The highest viscosity of okra mucilage increases the drag force in the laminar flow, in accordance with Stokes' law. This results in the lowest terminal velocity observed for the microparticles in okra mucilage.

**Table 2:** Terminal velocity (V_*s*_) & Maximum shear stress (*τ*_*max*_).

Material/Parameter	V_*s*_ (mm/s)	*τ*_*max*_ (Dyne/cm^2^)
Water	3.06E-01	0.123
PEO15 solution	5.10E-03	0.061
Okra mucilage	1.38E-03	0.110

For the aggregation of cell bulks, these mathematical relationships are obtained based on predictions from Eq. (1). When aggregation occurs, the terminal velocities increase exponentially with a rise in the radius. This leads to a significantly higher terminal velocity of microparticles in water compared to that in okra mucilage due to the low viscosity of water. A higher terminal velocity also leads to an exponential increase in shear, resulting in cellular damage and the disruption of cell aggregation [34]. As cell aggregates grow in size, higher rotation speeds are needed to provide sufficient shear conditions for their suspension. When the cell density surpasses that of the solution in the RWV system, the elevated rotation speed of the chamber accelerates the migration of aggregated cells towards the wall, thereby reducing the time required for cell collisions with the chamber wall [29], [28]. This wall effect causes damage to the cells. Higher viscosity solutions can decrease the rate of migration towards the chamber wall in aggregated cells. As a result, the high viscosity of okra mucilage improves adaptability to an increased cell aggregation radius. However, a decrease in the terminal velocity influences the mass transport of nutrients. The delivery of nutrient mass depends on the relative flow of nutrients and the movement of the cell, as well as on the diffusion rate of each solute in a particular culture medium [43], [44], [45]. Hence, In the future, a comprehensive study will be undertaken to elucidate the mass transfer coefficient of okra mucilage. Consider adjusting the amount of okra mucilage or employing pre-sheared okra mucilage as potential strategies to enhance mass transport.

When the Reynolds number is less than 1, the flow around a microparticle is characterized as creeping flow. The maximum shear stress is given by Eq. (2). Table 2 shows that the maximum shear stress is constant concerning the fluid viscosity and vessel speed. This is because the opposing effect of the viscosity on the relative velocity reduces its effect on the shear stress [34]. The microparticles in PEO15 exhibit the lowest value of maximum shear stress. The linear increase in maximum shear stress can be attributed to the proportional difference between the microparticle and liquid density [31], [46]. However, in both water and okra mucilage, the maximum shear stress of microparticles is similar at approximately 0.115dyne/cm^2^. The variation can be traced back to okra mucilage, a polysaccharide with a molecular weight of 1.9×10^6^(Da) [47]. In contrast, PEO15 exhibits a higher molecular weight ranging from 3.3 to 3.9×10^6^ Da. As the molecular weight of a polymer rises, there is a corresponding elevation in crystallinity, resulting in a subsequent increase in density. Therefore, the introduction of additional okra mucilage do not result in a notable impact on the physical density of the okra mucilage by the original solvent, it can offer a more controlled cellular environment.

### 3.6 Initial period

**Figure 8:**
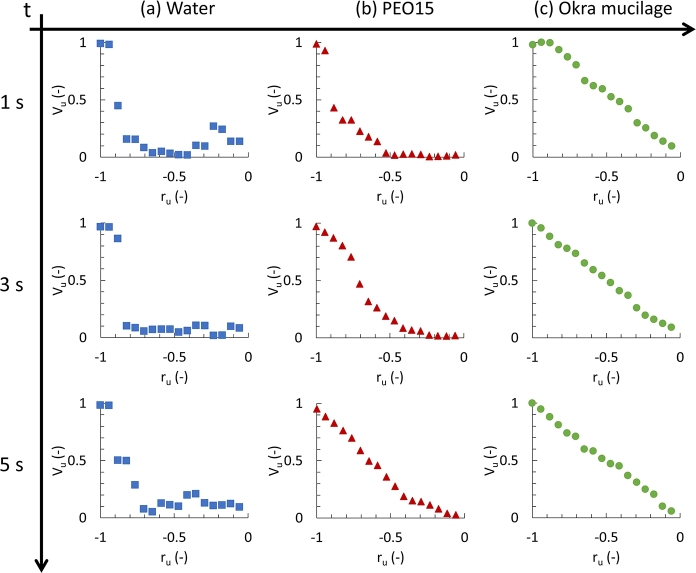
Velocity at unit radius −1 < *x* < 0 with time (5 s) in the initial stage; (a): Water, (b): PEO solution, (c): Okra mucilage.

Fig. 8 presents the velocity development information (0 – 5 s) of the microparticle in water (Fig. 8-a), PEO15 (Fig. 8-b), and okra mucilage (Fig. 8-c). In water, microparticles positioned near the wall demonstrate higher velocity and track the movements of the wall. The other portion involves microparticles with lower velocity distributed flatly along the radius within the range of -0.8 to 0. Thus, there is a noticeable dip in the water samples, indicating a sudden change in the curve. Over time, this distance gradually diminishes. In the PEO15 solution, the velocity of the microparticles increased slowly over time. At 5 s, the velocity curve of the microparticles exhibits a distribution close to a rigid-like rotation. However, in the okra mucilage sample, during the development time of 0 – 1 s, the outer three points near the wall display a plateau zone, while the remaining points decrease as the radius approached zero. By 3 s, the velocity profile exhibits a rigid-like rotation.

When the cylinder chamber with water began rotating, the outer layers near the wall experienced an increase in velocity due to the friction between the water and the cylinder wall. On the other hand, the fluid elements, located away from the wall, do not experience direct interaction with the wall. Consequently, their velocity initially remains close to zero. The observed phenomena can be attributed to the Newtonian properties of the fluid. When a sudden stress is applied to a purely viscous material system, strain lags behind stress. It suggested that water RWV systems require a long time to attain a balanced state.

In PEO15 solution and okra mucilage, the polymer (or polysaccharide) network is better transferring motion at the wall to the internal static fluid aligns with the behavior of viscoelastic materials that elastic elements cause an immediate stress response to the strain. It results in dynamic interactions between the wall and the internal fluid, influencing the behavior of fluid. When the cylinder wall starts rotating, the fluid near the wall experiences higher shear rates, leading to a decrease in viscosity in that region. This reduced viscosity allows the fluid near the wall to flow more easily. The combination of shear-thinning behavior and elastic response in the viscoelastic solution can lead to a more efficient momentum redistribution and alignment of polymer (or polysaccharide) chains, allowing the fluid to adjust to the rotation more rapidly.

Moreover, the occurrence of the plateau zone between 0 s and 1 s is attributed to the presence of an overshoot phenomenon of okra mucilage. The overshoot phenomenon occurs when a constant shear rate is suddenly applied to a viscoelastic material, causing the shear stress to increase rapidly with time until it reaches the maximum peak stress value and then gradually decreases to its steady state [48]. When the shear flow starts, entanglements among polymers (or polysaccharide) result in a temporary excess orientation of the sub-chains, and it will temporarily contribute to an elevation in both linear viscoelasticity and viscosity [49], [50]. This difficulty in flow near the wall indirectly leads to an increase in the wall thickness. Consequently, a plateau zone is observed in the velocity profile, extending from 0 to 1 s.

### 3.7 Final period

**Figure 9:**
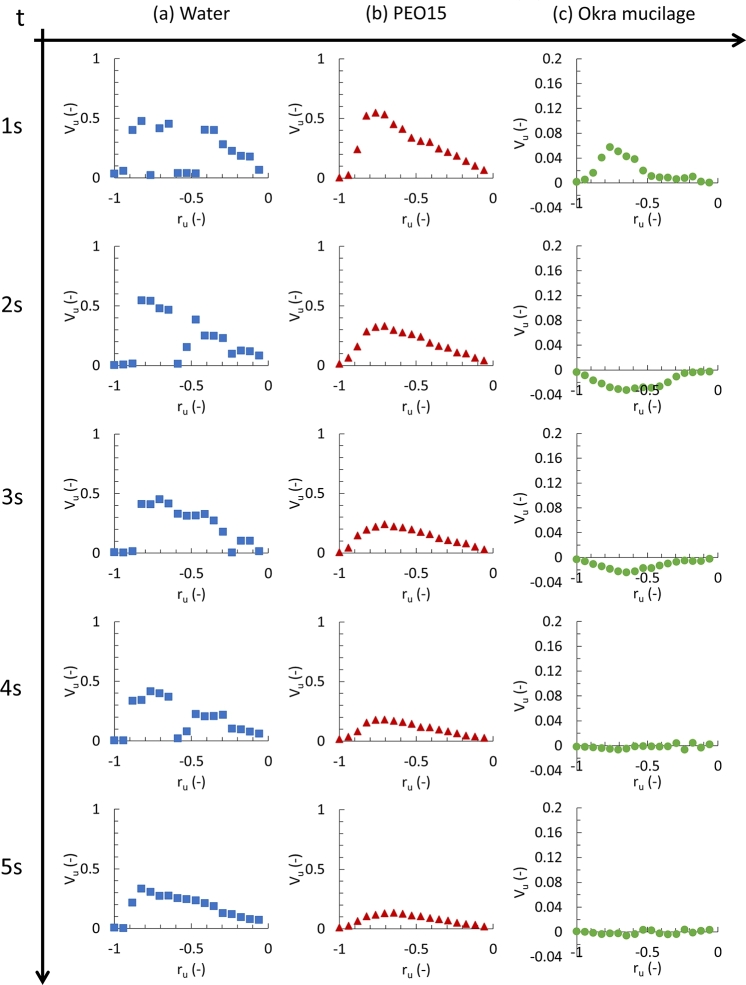
Velocity at unit radius −1 < *x* < 0 at various times (5 s) in the final stage; (a): Water, (b): PEO solution, (c): Okra mucilage.

Fig. 9 shows the velocity development at the end stage (0 - 5 s) of the microparticle movement in water (Fig. 9-a), PEO15 (Fig. 9-b), and okra mucilage (Fig. 9-c). In all the samples, the velocities near the wall are zero, following the boundary conditions. In the water, the velocity along the radius toward the chamber center exhibit instability, characterized by the presence of troughs. In the PEO15 solution, the microparticles experience a decrease in peak velocity, gradually moving toward the center over time. However, the velocity profile of okra mucilage exhibits a stark contrast to that of the other two samples. The relative velocity vector has the lowest value, and the negative values are found. It indicates a reversal in the flow direction. After 4 s, the velocity along the radius approached zero, bringing the system to a rapid halt.

In the water sample, when the horizontally rotating cylinder comes to an abrupt stop, microparticles near the wall collide at a high initial speed. Following the wall collision, the microparticles straighten into the interior area and collide with other microparticles. This may result in a reduction of detectable microparticles in the middle layer (between -1 to 0 unit length), posing challenges for PIV analysis. Furthermore, after 10 seconds, a phenomenon reminiscent of the tea-leaf paradox (a phenomenon in fluid dynamics where, after stirring a cup of tea, tea leaves move toward the center and bottom of the cup instead of being pushed to the edges as one might expect.) was observed in the water sample. This peculiar behavior is attributed to the sudden stop, where water flow near the front and back plates slows due to friction. The resulting secondary flow brings microparticles toward the center, contributing to the observed phenomenon. In the PEO15 solution, the presence of polymer networks restricts the movements of microparticles, eliminating the instability caused by the wall effect. The relative velocity gradually decreases over time.

However, in okra mucilage, after 1 s, the flow is reversed. This is because the okra mucilage storage modulus is dominant under 50 rpm (5.23 rad/s). This elastic reverse flow occurs when the Weissenberg number (elastic force/viscous force) was high (okra mucilage: *λ*=4.5 s; N1=0.02 Pa) [51]. The elastic reverse flow can be explained by a restretched structure [52]. The random coil structures fully develop in the flow direction while the system is running. As the RWV stops, the relaxed structure undergoes internal compression with decreasing velocity. Upon reaching this limit, the okra mucilage polysaccharides relax and restretch, creating a weaker but reversed flow. Moreover, when the elastic elements dominate over the viscous elements, the entire okra mucilage exhibits a quicker response to the cessation of the wall movement. The microparticles, having the lowest relative velocity, require less time to reach a balanced stage. But, the elastic reversed flow at the close-up stage may generate additional shear stress that could be detrimental to the cell spheroids. It is recommended to decrease the rotation speed gradually to prevent elastic reversed flow and protect the cell spheroids from additional shear stress injury in future experiments.

## 4 Conclusion

This study provides a detailed analysis of the flow structure of okra mucilage rotating in the RWV system from 1 to 50 rpm. The results obtained for the okra mucilage are compared to those for PEO15 (an artificial chemical non-Newtonian fluid) and water (a Newtonian fluid): 1. The flow structure of the okra mucilage in the RWV system displayed concentric circular rigid-like rotation from 1 to 50 rpm. 2. The terminal velocity of microparticles in okra mucilage decreases while maintaining a maximum shear stress similar to that of the solvent (water). 3. Okra mucilage responds quickly to rotational movement from the start of the motion. Moreover, the weak elastic reversed flow occurs at the close-up stage because the elastic modulus is higher than the storage modulus. Our findings highlight the relationship between the flow structure of okra mucilage and its rheological properties in the RWV system.

However, adding okra mucilage will also expose some other problems, such as influenced nutrient transfer due to the viscosity increases, and elastic reverse flow at the final period. These will be improved in the future by quantifying the amount of okra mucilage and changing the startup methods in the biological research stage related to cells.

## Funding statement

The authors acknowledge the financial support provided by the Tokyo Research Initiative for Sustainability at 10.13039/501100010236Tokyo Metropolitan University during the preparation of this manuscript.

## CRediT authorship contribution statement

**Weijun Zhu:** Writing – review & editing, Writing – original draft, Visualization, Validation, Methodology, Investigation, Formal analysis, Conceptualization. **Hiromichi Obara:** Writing – review & editing, Supervision, Project administration, Funding acquisition.

## Declaration of Competing Interest

The authors declare that they have no known competing financial interests or personal relationships that could have appeared to influence the work reported in this paper.

## Data Availability

Data will be made available on request.
